# Analyzing the Local Epidemiological Profile of Malaria Transmission in the Brazilian Amazon Between 2010 and 2015

**DOI:** 10.1371/currents.outbreaks.8f23fe5f0c2052bfaaa648e6931e4e1a

**Published:** 2018-03-27

**Authors:** Tiago Canelas, Carlos Castillo-Salgado, Helena Ribeiro

**Affiliations:** School of Public Health, University of São Paulo, São Paulo, Brazil; Department of Epidemiology, Johns Hopkins Bloomberg School of Public Health, Baltimore, MD, US.; School of Public Health, University of São Paulo, São Paulo, Brazil

**Keywords:** Brazil, Epidemiology, malaria, spatiotemporal distribution, transmission

## Abstract

**Introduction:**

Malaria still is a public health problem in the Americas. In 2015, Brazil accounted for 37% of all cases in the Americas, and of these cases, 99.5% were located in the Brazilian Amazon. Despite the mobilization of resources from the Brazilian National Plan for Malaria Control, too many municipalities have high transmission levels. The objective of this study is to evaluate the local epidemiological profile of malaria and its trend between 2010 and 2015 in the Brazilian Amazon. This study also aims to recognize the epidemiological differences in the local temporo-spatial dynamics of malaria.

**Methods:**

Malaria data were stratified by the annual parasite incidence (API) over the six-year period and by municipality. We used the method of seasonal decomposition by Loess smoothing to capture trend, seasonal and irregular components. A generalized linear model was applied to quantify trends, and the Kruskal-Wallis Rank Sum was applied to test for seasonality significance.

**Results:**

The malaria API declined by 61% from 2010 to 2015, and there was a 40% reduction of municipalities with high transmission (determined as an API higher than 50). In 2015, 9.4% of municipalities had high transmission and included 62.8% of the total cases. The time-series analyses showed different incidence patterns by region after 2012; several states have minimized the effect of the seasonality in their incidence rates, thus achieving low rates of incidence. There were 13 municipalities with sustained high transmission that have become the principal focus of malaria control; these municipalities contained 40% of the cases between 2013 and 2015.

**Discussion:**

Brazil has achieved advances, but more sustained efforts are necessary to contain malaria resurgence. The use of malaria stratification has been demonstrated as a relevant tool to plan malaria programs more efficiently, and spatiotemporal analysis corroborates the idea that implementing any intervention in malaria should be stratified by time to interpret tendencies and by space to understand the local dynamics of the disease.

## INTRODUCTION

Despite the huge advances towards the reduction and elimination of malaria, Brazil still had a significant malaria problem in 2018. The efforts of Brazil to achieve a 75% reduction in the case incidence rate between 2000 and 2015, following the United Nations Millennial Development Goals (MDGs) target 6C, were remarkable. With 143,549 cases in 2015, Brazil reached an 89% reduction since the beginning of the MDGs 1.Despite this achievement, in 2016, Brazil had 18% of all malaria cases that were confirmed by the World Health Organization (WHO) for the region of the Americas 2, and 99.5% of the Brazilian cases were in the Legal Amazon, encompassing the Brazilian Amazon forest^3^.

The Brazilian Institute of Geography and Statistics (IBGE) delimitated the so-called Legal Amazon in 1953, which included nine states (Acre, Amapá, Amazonas, Maranhão, Mato Grosso do Sul, Pará, Rondônia, Roraima and Tocantins) and encompassed 772 of their municipalities^4^. Nonetheless, the Amazon region is vast and diverse. In 2015, our study site comprised 7.83% of the total population of Brazil and 42% of the territory. However, there are disparities between states; for example, Amazonas and Pará are the largest states, and together, they represent 76% of the population and 66% of the municipalities of the area studied. Both are the poorest states, with 234 US$ and 209 US$ per capita of monthly income, respectively. However, internal disparities are present within the states. In Pará, the municipality with the highest income per capita is Canaã dos Carajás, with 3,000 US$ per month, whereas the poorest, Curralinho, has only 90 US$^4^. Thus, this area is highly heterogeneous with a complex ecology and different socioeconomic characteristics, making the study of internal dynamics extremely important to prevent, control and eliminate local malaria^5^.

Brazil has had a predominance of the transmission of the *Plasmodium vivax* parasite since the 1990s^3^^,^^6^ and accounted for approximately 87% of total cases in 2016 7. Although the National Malaria Prevention and Control Programme (NMCP) has specific aims to control *Plasmodium falciparum* due to its deadliness, attention has been growing for *P. *vivax recently because it is more difficult to control compared to *P. falciparum*, particularly due to its relapses, lower parasitemia and resilience, which add complexity to prevention and control interventions 3^,^^8^^,^^9^.

Brazil has a long history of fighting malaria. In the past, endemic areas were found throughout the country, but through successive programs (National Malaria Service, Malaria Eradication Strategy by WHO), the endemic areas shrunk toward the Amazon Region 3^,^^6^. This region experienced a dramatic population immigration that was immunologically naïve to the parasite between 1970 and 1990 3^,^^10^; consequently, the number of malaria cases increased to levels that the region had not experienced in a long time 3. Given that most cases occurred in the Amazon forest, the Brazilian government launched three programs with a special focus on the region with the objective of significantly reducing the number of cases 3^,^^9^. The NMCP finished in 2015, and the Ministry of Health launched the new Plan for the Elimination of Malaria in Brazil 11, supported by the Global Technical Strategy of WHO and Roll Back Malaria for 2016 – 2030, as a part of the Sustainable Developed Goals, to end the malaria epidemic by 2030 12^,^^13^

Epidemiological stratification has been identified as a valuable tool to achieve prevention, control or even a plausible elimination in the region of the Americas 14. That, in conjunction with the organized and synergic use of multiple strategies, can foster better results in malaria programs 15^,^^16^. Therefore, the main objective of this study was to evaluate the local epidemiological profile of malaria and its trend between 2010 and 2015 in the Brazilian Amazon Region. Furthermore, this study aimed to recognize the epidemiological differences in the local temporo-spatial dynamics of malaria in this region.

## METHODOLOGY

The area of study is the Legal Amazon (figure 1). Due to the lower number of malaria cases, the states of Maranhão, Mato Grosso do Sul and Tocatins were discarded, leaving 310 municipalities for the study in the remaining 6 states, all of which are located in the Brazilian Northern region. The analysis excluded the municipality of Mojuí dos Campos because the municipality boundaries were redrawn from other municipality in 2013, and there was no information available for most years.

**Map of the study site. Elaborated by the authors:**
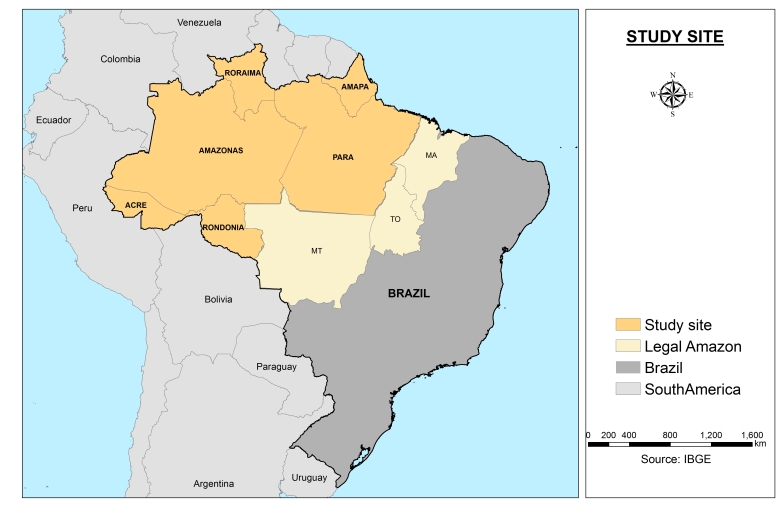

Malaria cases from 2010 to 2015 were obtained from the Epidemiological Surveillance Information System (SIVEP-Malaria), a database implemented in 2003. Despite the difficulties presented in the study site, the database performed very well 6. For the variable “municipality of notification” of the malaria case, the database had 100% completeness between 2010 and 2012; however, because it is a real-time database, some delays have been reported, depending on Internet availability 17. Since the aim was to understand malaria transmission within the Brazilian Amazon, we extracted all monthly autochthonous cases of all parasites with confirmation made by local health professionals by Rapid Diagnostic Tests (RDTs) and thick blood smears after diagnosis 17 and excluded the imported cases. We calculated the Annual Parasite Incidence (API) (confirmed number of positive slides for parasite over 1 year for the population under surveillance x 1000) and the monthly incidence in each of the 310 municipalities. Population data were obtained from the 2010 National census, and IBGE estimates were used for the years 2011 to 2015.

**ANALYSIS**

We described a time series of malaria incidence for the 72 months of study for the entire area and divided by state. To address irregularities and error components, we smoothed the time series using a centred moving average of k=5, in which k is the number of observations that are averaged. This value was selected because it highlighted the best pattern of the data. We used the method of seasonal decomposition by Loess smoothing to capture the trend, seasonal and irregular components. A generalized linear model was applied to quantify this overall trend, and the Kruskal-Wallis Rank Sum test was applied to test for seasonality significance.

There are differences between the thresholds of what is considered high transmission malaria, depending on the region in the world 18. For this study, we followed the guidelines of the Ministry of Health of Brazil for malaria transmission, categorizing malaria transmission into 3 stratums: low (<10 API), medium (10 – 50 API) and high (>50 API) 9. Since most of the municipalities had a very low API (less than 1 or 0), we decided to analyze and depict our data using those municipalities with the higher burden of disease to identify different local patterns. To evaluate these municipalities, we selected the high transmission municipalities per year for the period 2010 to 2015.

## RESULTS

By extracting the data from the SIVEP-Malaria, we were able to characterize the epidemiological profile from 2010 to 2015. Figure 2 shows the decrease of autochthonous cases from the highest number of cases (32,567 in July 2010) to the lowest number (8,122 in February 2015). The smoothed line helps to clearly perceive the seasonal trend in the central months of each year (June to August). A quantification of the seasonal trend was made by the Kruskal-Wallis Rank Sum test, which showed statistical significance (p-value < 0.05), suggesting an increase of more than 15% of cases in the peak months and a decrease around the same percentage in the last months of the year and the beginning of the following year. In the axis on the right, we observed the tendency of API over time to decrease significantly from 2010, when it was 21.54, to 2015, when it was 8.38. The blue dotted line is the target of the NMCP to decrease the API for the Legal Amazon to 9.45 by the end of 2015. At the beginning of 2014, the number of cases was already lower than the target. As mentioned, our study does not include three states of the Legal Amazon (Maranhão, Tocantins and Mato Grosso do Sul), but adding these states to our analysis would lower the API for the region for the entire period; thus, in 2012, the region would have achieved the goal of an API of 8.63 (data not shown).

**Number of autochthonous cases and yearly API in the study site between 2010 - 2015:**
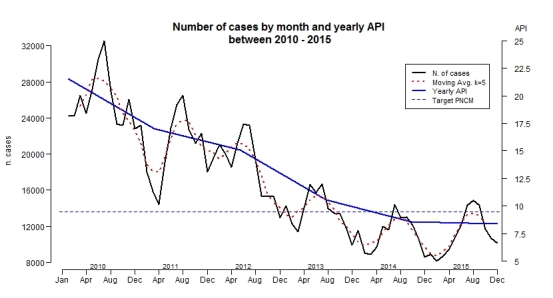

Figure 2 shows the big picture in the entire area, albeit differences were found between regions, states and municipalities. In table 1, we stratified malaria by region, state, study site and the entire Legal Amazon for the year 2015 by comparing the API of every part to the NMCP goal. When stratified by state, only Pará and Rondônia met the target; the other states were far from achieving it, particularly Acre, with an API of 33.1 in 2015. For this reason, it is important to analyze the local differences in the dynamics of transmission by month, per season and per state.

**Stratification of Annual Parasite Incidence, autochthonous cases and population in 2015 by region and states:** 

Region or State	API 2015	Autochthonous cases	Population 2015
Target NMCP 2015	9.45	-	-
Study site	8.38	134,049	15,989,320
Legal Amazon	5.15	143,129	27,781,789
Acre	33.10	26,595	803,513
Amazonas	18.58	73,174	3,938,336
Amapá	16.91	12,968	766,679
Roraima	12.12	6,131	505,665
Rondônia	3.57	6,381	1,768,204
Pará	1.07	8,800	8,206,923

Looking individually at each state, the outcomes are very heterogeneous, aligned with the results of Lima et al. 19. Figure 3 depicts the monthly incidence for each state in the study period and the smooth moving average for 5 months. We found two different groups: those that had a negative tendency (Pará (PA), Rondônia (RO) and Roraima (RR) named as (PRR)), and those with a stationary tendency (Acre (AC), Amazonas (AM) and Amapá (AP) named as(AAA)). Those states with a negative tendency did not present high seasonality in the incidence since 2012; instead, they showed different local malaria patterns. In figure 3 and table 1, we observed that in PA and RO, the API and monthly incidence continued to decrease since 2012, a fact that helped them achieve the target of the NMCP. Roraima is on its way to hit the target as well, whereas AC, AM and AP have much work to do to achieve this target.

**Incidence by month and State between 2010 and 2015 (black line). Moving average of 5 months (slash red line).**
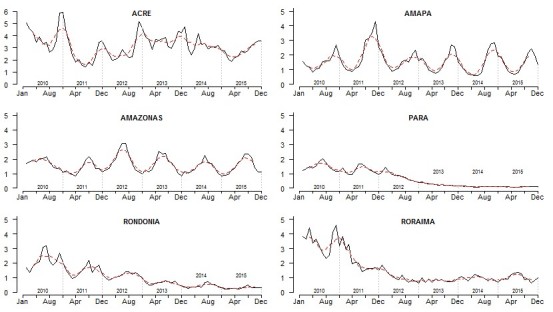

We found statistically significant (p-value < 0.05) different malaria transmission peaks for each state (figure 4). However, for the states of PA, RO and RR, the seasonality was not statistically significant, as shown in figure 3. In figure 4, the months highlighted indicate the peak of malaria transmission in all years. As the table illustrates, the malaria transmission peak is not coincident in all the states in any single month. We observed that January and June to September present peaks of malaria transmission in four states. Only April was not a peak month in any state. The relevance here is the heterogeneity of malaria transmission peaks in the 6 states of the Amazon region, indicating that local control interventions relevant to the time and place are required.

**Highlighted cells represent the months of malaria transmission peaks by States:**
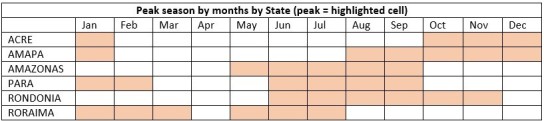

Analyzing all the municipalities, we observed that the epidemiological profiles differed between the AAA and PRR groups (figure 5). For the years 2010-11, the profile appears unpredictable with several ups and downs; however, the incidence changes after 2012 for both groups. For AAA, there was clear seasonality and stationarity (both p-value < 0.05) with a very pronounced increase in incidence in the middle of each year. Conversely, in PRR, there was a marked decrease (p-value < 0.001) in the incidence and a remarkable smoothing in the seasonality; however, seasonality was still present (p-value < 0.001). Despite the clear differences in the epidemiological profile of both groups, similarities might be found when examining the municipalities with the highest burden of malaria.

**Monthly incidence of malaria for both groups; AAA and PRR, 2010 - 2015:**
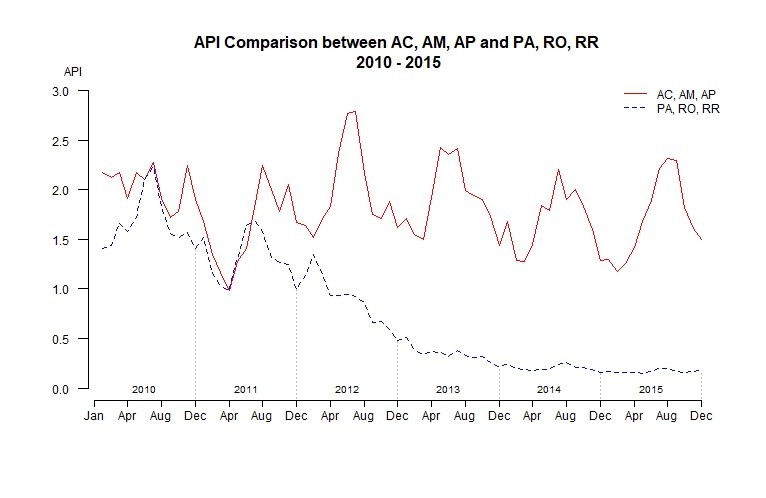

Table 2 presents the number of municipalities, per state, with an API higher than 50. In 2015, Rondônia was the only state without any municipality with high transmission. The AAA states did not show a decreasing number of municipalities with high transmission. In fact, Acre and Amazonas had increases in the number of high transmission municipalities from 2014 to 2015. Conversely, PRR took great strides toward decreasing high transmission municipalities.

**Number of municipalities with Annual Parasite Incidence, 2010 – 2015.** 

		Number of municipalities with API equal or higher than 50				
	2010	2011	2012	2013	2014	2015
ACRE	5	3	3	3	3	4
AMAPÁ	4	6	6	4	5	4
AMAZONAS	10	9	19	19	14	17
PARÁ	13	13	10	4	1	1
RONDÔNIA	7	3	1	1	1	0
RORAIMA	10	5	2	2	2	3
Total municipalities	49	39	41	33	26	29
% of the total cases	66.8%	58.2%	63.2%	66.7%	58.6%	62.8%
% of the total cases in sustained municipalities	25.5%	22.6%	30.9%	37.2%	40.1%	39.8%

Figure 6 shows those municipalities with the sustained high transmission in the period of 2010 – 2015. We present this information in the last row of table 2, which shows the percentage of total cases that occurred each year. Over time, these 13 municipalities with sustained high transmission rates have become the principal focus of malaria in the study area, containing almost 40% of the cases between 2013 and 2015. It is important to highlight the fact that seven of these municipalities are located on the border with other countries. However, in our study, only autochthonous cases were considered, so we did not consider the possible importance of imported cases in the spread of the disease.

**Map of the municipalities with sustained high transmission from 2010 to 2015.**
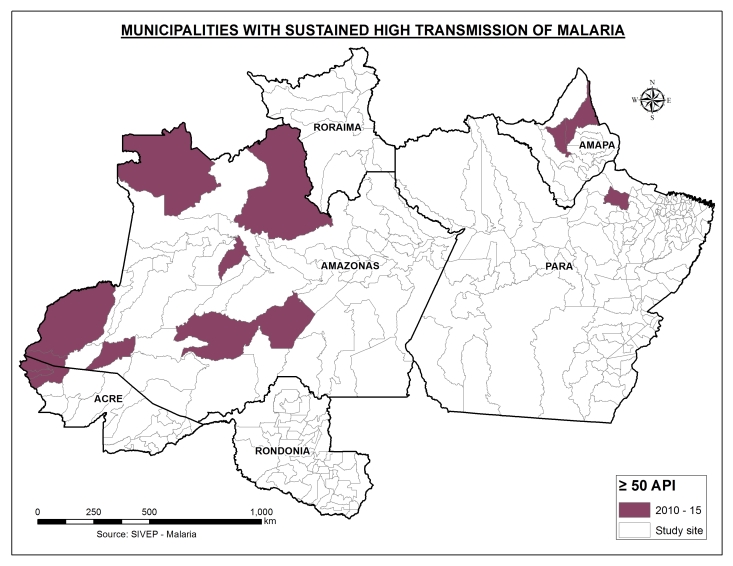

To select the complementary priority municipalities, we included the municipalities with high transmission in each year (figure 7). In dark red are the municipalities with very high transmission, higher than 100 API, and the light red these with an API between 50 and 100. Anajás in the state of Pará was, between 2010 and 2012, the municipality with higher incidence of malaria, with a peak of 915 API in 2010 In 2013 – 2014 Manâcio Lima in Acre had the highest API, and in 2015, Serra do Navio in the state of Amapá had the highest incidence, with an API of 343. These municipalities represented at least 58% of the total autochthonous cases annually, reaching 66.8% in 2010, as presented in table 2 For example, in 2014, only 26 municipalities contained 58.6% of all cases. Since 2010, there were reductions in 20 high transmission municipalities. In addition, the number of municipalities with zero cases increased from 11 municipalities in 2010 to 76 in 2015 (table 2).

**Map of the 50 municipalities with the highest Annual Parasite Incidence in 2015.**
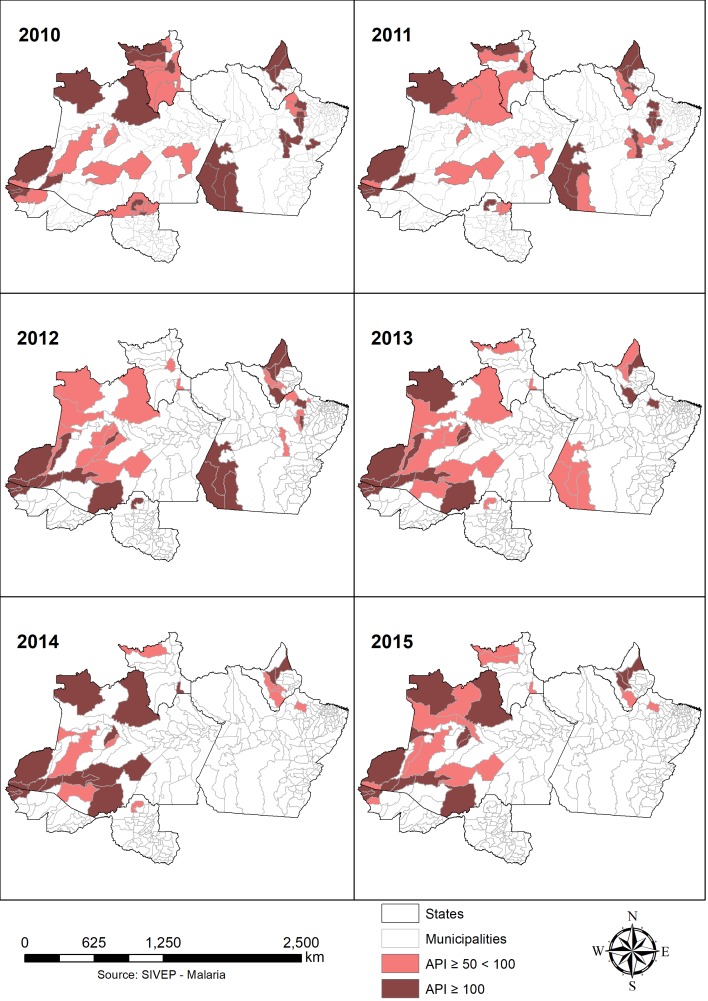

We have seen the dynamics of malaria API for the entire study area and by state; however, a temporal trend description was necessary. Figure 8 depicts the median incidence per month in the high transmission municipalities and the median incidence by group of states. The median incidence per month among the municipalities with high transmission each year presents a flat tendency but not statistically significant. The top graph of figure 8 shows a slightly negative tendency overall, but there was an irregular pattern, with an increase from 2014 to 2015. The Kruskal-Wallis test for seasonality was statistically significant (p-value < 0.05), with the peak of transmission in the middle of the year, and low transmission occurring at the end and beginning of the year. This pattern becomes clearer from 2012 onwards. Due to the information provided in the previous sections, we decided to disaggregate these high transmission municipalities in the two previously mentioned groups: AAA and PRR. In the bottom graph of figure 8, we can identify two dynamics: AAA had a stationary tendency, with a slight increase in the incidence in the last four years. The seasonal trend is also clearer from 2012 onwards. Conversely, the municipalities of PRR displayed a very different dynamic. From 2010 to 2012, there was a strong decrease in the incidence from a peak of 23.59 in March 2010 to 2.77 in October 2015. However, the most important characteristic is the stabilized low incidence since 2013 with little variation between months.

**Average monthly incidence in the 50 municipalities with higher transmission and average monthly incidence by groups of states:**
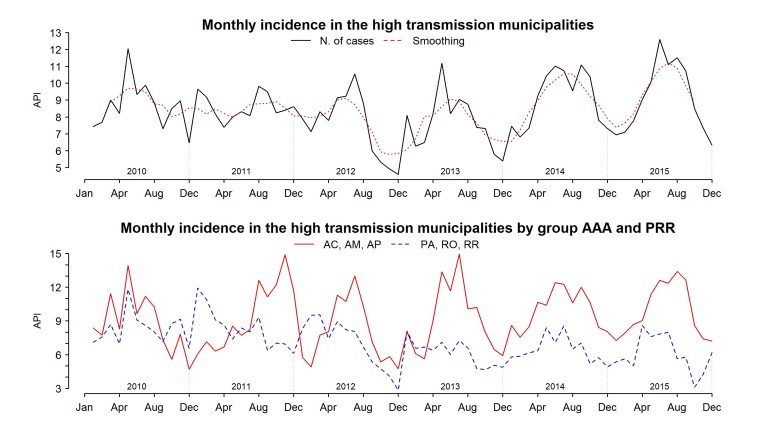

Regarding parasite prevalence, *P. *vivax was predominant with 88.5% of the cases in 2015. Although *P. falciparum* represented only 10.93% of the cases in 2015 (the rest were P. ovale and P. malariae), it is important to target *P. falciparum* because it leads to the most complicated malaria, and the mortality rate follows the dynamics of *falciparum* cases 3. The dramatic decrease of *falciparum* has resulted in a very low number of deaths. In the study site, there were 16 deaths in 2014 (data from SIVEP-Malaria). For the 29 high transmission municipalities in 2015, there were 11.160 cases of *P. falciparum*, representing 53.1% of the total burden in that year. When we look at the municipalities with the sustainable high transmission, the 13 municipalities had 39.4% of the total cases. However, only three municipalities (Cruzeiro do Sul, Mânacio Lima and Rodrigues Alves) in the state of Acre accounted for 24.64% of the total *P. falciparum* cases.

## DISCUSSION

It is clear the substantial advances toward malaria elimination in Brazil. This study shows the remarkable decreasing trend over the last six years, in concordance and as continuity of the results presented at state level by 19 for the period 2004 - 2013. In 2014, Brazil reported its lowest number of cases in 35 years 3^,^^20^, reaching the UN MDG and Brazilian’s goals for 2015 1^,^^5^, although a slight increase was observed in 2015. The Brazilian national policy for malaria might have played a central role since investing in community human resources is essential to plan interventions. Investing resources not only at the state level but also at the municipal level has targeted those municipalities with the highest risk of malaria transmission, thus improving the interventions. Despite all these efforts, Brazil must maintain its interventions against malaria to face the challenges of symptomless infections, anti-malarial drug resistance, vector behaviour, environmental changes and border malaria 3^,^^20^, due to the resurgence, instability and persistence of an elevated number of cases in some municipalities, as shown in this paper. A possible shortage of resources in the new malaria policy for 2016 – 2030 might dismantle the progress made until now.

This study demonstrated that it is possible to stabilize malaria in low transmission areas in the Brazilian Amazon. This is relevant because this stabilization has minimized the effect of seasonality, which remains a large problem for municipalities with high transmission in the states of Acre, Amapá and Amazonas. Notwithstanding, very low transmission might mask seasonal patterns, requiring improvements in surveillance 21. As transmission declines, interventions should be adapted to the new malaria typology 22. There are several municipalities with high transmission, whereas others nearby do not have high transmission. These results indicate geographic and temporal differences that might be explained by the influence of the interventions upon the risk factors of the disease. They also suggest the need to establish evidence-based, integrated and specific control strategies in order to tackle the local transmission in concordance with the results of 19.

Local interventions should have different goals: for the low transmission areas. Understanding the differences that contribute to the continued transmission should be the priority in order to apply a more comprehensive strategy to attack the pockets of clinical malaria by identifying population groups that are more susceptible, with special attention to malaria in pregnancy, asymptomatic infections, providing diagnostics and treatment directly and enhancing surveillance^12^^,^^20^^,^^21^^,^^23^. In regions with higher transmission and seasonal patterns, interventions should focus on lowering the number of cases in the peak season and on stabilizing transmission in the other months. This might be achieved following the Global Technical Strategy 12 of specific vector control actions using local data, scaling up diagnostic testing and treatment, using locally specific recommendations as the diagnostic of G6PD deficiency, providing new treatment schemes for these patients, and building up strong networks to fight against malaria, as also suggested by 3^,^^20^.

However, despite efficient interventions, there are some exceptions. Those exceptions are areas with stabilized low malaria transmission that present some municipalities with a very high API, such as Anajás in PA, which has had high transmission levels for at least the last six years; or municipalities in Roraima, with APIs greater than 100. In Roraima for instance, the presence of vectors, other than An. Darlingi, require more research in mosquito populations´ in order to suit vector control strategies 19^,^^24^. These municipalities require further investigations and local analysis to understand the specific risk factors that are influencing them to have a much higher transmission than their neighbours. Although there is no consensus on the influence of the risk factors in transmission, there are numerous factors that make transmission plausible 25^,^^26^^,^^27^. Precipitation and temperature are some of the more recurrent variables in the literature 25^,^^27^ and follow a clear seasonality (figure 3 – 4) that might explain the cycles of transmission. The changes in land use and land cover, new settlements, the presence of mining (i.e., gold), fish ponds or deforestation might also explain the high transmission in some municipalities 3^,^^20^^,^^25^^,^^26^^,^^28^.Environmental changes that affect the ecosystem, such as deforestation or new settlements, have been shown to foster an increase in the number of cases 3^,^^10^^,^^20^^,^^25^^,^^26^. Financial constraints also might change the outcome of the interventions; as high-risk municipalities have the right to more resources, lowering this risk might compromise their budget 9, possibly causing a conflict of interest for local authorities. Lowering transmission might mean receiving less money.

The municipalities with sustained malaria (figure 6) are the key municipalities where resources should be applied to obtain better results in the control and elimination programs. Nevertheless, we believe that authorities should target more than these 13 municipalities to achieve a permanent low level of cases. Because of that, targeting the 50 municipalities with the highest transmission (figure 7) could be more efficient. These areas have the potential of reducing the burden of disease by almost 40% in the following years; however, to achieve that, more local (geographically and methodological) interventions should be adopted by the authorities. The reduction of municipalities with high transmission has occurred from 2013 onwards; therefore, understanding which strategies have contributed to this decrease is crucial to consolidate and improve actions 6. For example, one strategy that was implemented after a success in Acre in 2006 was to provide long-lasting insecticide-treated bed nets (LLINs) to all the municipalities in the Amazon Region between 2009 and 2011 3^,^^5^^,^^29^. Conversely, another study showed no effect in the decrease of API with LLINs in Rondônia 30. Although Brazil has provided LLINs and insecticide-treated nets free of charge since 2007, only approximately 30% of the region is covered, and the success of these tools is difficult to establish due to the lack of studies of systematic evaluation of interventions on the vectors in the region 8^,^^12^^,^^20^^,^^30^^,^^31^. The ecology and the vector behaviours in the region are not well understood because of the lack of studies and the regional variations 20. In the study area, the predominant mosquito is Anopheles Darlingi 6^,^^31^^,^^32^. It is a vector that lives both in modified environmental settings made by humans, i.e. fish ponds, and natural rural places such as rivers 3^,^^10^^,^^28^^,^^32^^,^^33^. There are several studies that highlight how this vector is highly adaptive to human behaviour and varies from place to place 32. Although some studies have corroborated an exophagic and peri-domestic behaviour 31^,^^34^, others have shown an intra-domestic, endophagic behaviour 30^,^^31^. Several regions that have switched the local epidemiology of malaria and dramatically reduced the incidence, such as the states of Rondônia and Roraima, should be analyzed by the authorities to obtain public health evidence upon which to base upcoming policies and interventions.

As mentioned in paper 35, we observed different territorial aggregation in figure 7. There are inter-municipality aggregations, particularly in AM, and inter-state aggregations, from west AC to north RR. In 2015, 5.34% of the cases in the study region were imported from other states. Although this study only considers autochthonous cases, internal migrations from regions with low exposure to malaria to areas with higher exposure have been associated with the increase of malaria in other periods of time in Brazil 3^,^^10^^,^^36^. In addition, third-level aggregations are located in the boundaries with other countries. Notably relevant are the countries with a major contribution in the number of cases in 2015 in the Americas region: Venezuela (30%), Peru (19%) and Colombia (10%), representing with Brazil the 83% of all cases in the Americas in 2015 7 and contributing to 4,966 cases in Brazil 1. Exceptionally alarming is the case of Venezuela, where a lack of governance in the health systems, shortage of antimalarials, few control measures and low budget in the malaria national plan have resulted in a dramatic increase of cases, including *P. falciparum*, and contributing to approximately 78% of the imported Brazilian cases in 2015 20^,^^37^^,^^38^. These three-level aggregates add an extra layer of complexity to a very complex disease because they indicate that interventions should be coordinated, not only at the municipality level, where most of the actions occur 9^,^^35^, but also at the interstate level and, most difficult, at a regional international level. As mentioned by 20 in the past, regional networking initiatives such as the Amazon Malaria Initiative (AMI) or the Amazon Network for the Surveillance of Anti-malarial Drug Resistance (RAVREDA) have contributed to policy change and to malaria control. In the time of malaria elimination, these initiatives should be strengthened 39.

Although this study did not discern between parasite species, it is important to plan interventions for different parasites because they present different patterns and epidemiology as presented by 19. Little attention was given to *P. *vivax until recently, but tackling this parasite is indispensable for malaria elimination to succeed 12^,^^20^. Diverse challenges to malaria prevention and control in the Amazon forest were highlighted by different authors 3^,^^12^^,^^20^^,^^40^; asymptomatic and sub-microscopy malaria (SM) infections that are more common in places with low transmission and where malaria elimination is being targeted present a significant challenge to control activities 41. How to detect asymptomatic malaria and SM are crucial to minimize the dissemination of new infections. Despite the large increase of health centers, technicians and microscopes, in most cases, SM is undetectable without PCR 42. Facing this situation, local health authorities and governments should have the flexibility to target these areas with residual malaria and look for asymptomatic or SM cases. Although it is not associated with complicated malaria, some studies have shown severe malaria with *P. *vivax in the study site 6^,^^43^ and others 44^,^^45^. Chloroquine (CQ) resistance in the Brazilian Amazon is the main problem for the treatment of *P. *vivax. This presents the problem of a neglected potentially severe disease with large concerns for public health authorities 20. Thus, more studies in the Amazon region are necessary because there is a lack of studies working with the local epidemiology of *P. *vivax to analyze the large patterns that are essential for policy-makers 46.

Because natural immunity for *P. falciparum* is not acquired in this region due to the low exposure to this particular parasite 9, the following three municipalities deserve special attention: Cruzeiro do Sul, Mânacio Lima and Rodrigues Alves. They have been a persistent malaria focus 47 since the epidemic of 2004-2006 related to proximity to fish farming, deforestation, closeness to the forest and internal migrations 5^,^^19^^,^^28^^,^^46^. All three municipalities border Peru, therefore, authorities should look at possible cases importation, in spite of the limited movement across the border. Regarding that, the NMCP strengthened efforts to decrease the number of* P. falciparum* cases and reduce the number of deaths due to malaria, which is one of the main objectives of the National Policy 3^,^^9^. Indeed, the new Plan for the Elimination of Malaria in Brazil focuses highly on* P. falciparum* with the goal to eliminate it from the region 3^,^^11^ by working closely with all countries of the Amazon region and regional initiatives such as RAVREDA and AMI. The aim is to avoid the possibility of resistance to artemisinin derivatives that might circulate in areas of Suriname, Guyana and French Guyana 3. In addition, recent attention for Plasmodium malariae has arisen due to some studies suggesting that its real incidence might be underestimated due to the difficulties to discern this parasite from *P. *vivax 20.

Difference between urban and rural, and road or riverine accessible settings, was not possible to highlight in this study because cases were identified only by the municipality of notification. Although the changes in malaria risk between these settings might be unclear, gradients between these areas should be incorporated into the analysis for proper stratification in future studies as suggested by 48. Traditional dichotomization between rural and urban might be inappropriate since some areas with high risk of transmission are located in the limits between these categories, as peri-urban malaria, and the risk of misclassification might lead to taking wrong interventions 3^,^^5^^,^^48^.

Some limitations of this study are due to its nature: the scale of analysis in a vast region with a high heterogeneity in the epidemiological profile and in the environmental and socioeconomic settings. Furthermore, the use of political boundaries might mask the real heterogeneity and dynamics of the disease as the natural boundaries of malaria and population are not coincident. In addition, this analysis did not differentiate between rural and urban malaria, and that might imply extensive changes in the prevention and control strategies 49 and in different local epidemiological profiles between and within municipalities.

## CONCLUSION

Notwithstanding the little attention given recently to malaria epidemiology, to understand the local epidemiology in population, time and space in a greatly heterogeneous territory is of vital importance for malaria prevention, control and elimination in the region of the Americas. The use of malaria stratification is a relevant tool and should be used as a key strategy to plan malaria programs more efficiently and effectively. These plans are set at the national level but with the flexibility to lower levels to articulate interventions based on their needs. Therefore, this study adds a value when assessing the whole area but stratifies by municipality allowing to see the importance of the local interventions in different epidemiological settings. Through spatiotemporal analysis, the study corroborates the approach that implementing any intervention in malaria should be stratified in time to interpret the latest tendencies and in space to understand the local dynamics of the disease. Geographic Information Systems and analytical mapping should be used as tools to help plan interventions with more accuracy. Brazil has achieved remarkable advances, but more sustained efforts at all levels are necessary to contain malaria resurgence. There are examples of successful interventions in the Amazon region that should be taken into account for future national policies. These interventions have helped several municipalities and states to stabilize the impact of the seasonality of malaria transmission and to reduce the number of new cases and the overall API. This sets a baseline for other municipalities and states, or even neighbour countries in the Amazon region, to adapt interventions to their epidemiological reality, without taking boundaries as the delimiting area of action to achieve a stable and sustained decrease of malaria transmission. The Amazon region must also strengthen collaboration with other countries since most of the critical areas of transmission are by international borders. Perhaps malaria areas of interest should be established by all the countries in the Amazon region to make joint efforts and turn interventions more efficiently. The Amazon region should be a priority in the Americas, and Brazil should maintain its efforts to decrease the number of cases. In concordance with the Global Technical Strategy, malaria should be a technical, economic, and political priority because, as is presented in this study, whenever commitment exists, malaria is controllable and preventable, leading to an important improvement in public health.

## Corresponding Author

Tiago Canelas at tcanelfe@usp.br

## Funding

Tiago Canelas acknowledges a predoctoral grant from the Government of Andorra, ATC014-AND-2015/2017 and the scholarship by the Coordination for the Improvement of the Higher Level Personnel (CAPES), Ministry of Education, Brazil. The funders had no role in study design, data collection and analysis, decision to publish, or preparation of the manuscript.

## Competing Interests

Tiago Canelas, on behalf of all the authors of the manuscript submitted to PLoS Current Outbreaks DECLARE that no competing interests exist.

## Data Availability

Access in figshare: https://figshare.com/s/eb5f1e1bea7c55bb7d94 DOI: 10.6084/m9.figshare.5923660

## References

[1] Ministério da Saúde. Malária |Situação Epidemiológica - Dados. 2018. [cited 2018 Jan 17]

[2] WHO. World Malaria Report. 2017

[3] Ferreira MU, Castro MC. Challenges for malaria elimination in Brazil. Malar J. 2016 May 20;15(1):284. PubMed PMID:27206924. 27206924 10.1186/s12936-016-1335-1 PMC4875681

[4] IBGE :: Instituto Brasileiro de Geografia e Estatística. 2017

[5] Costa KM, de Almeida WA, Magalhães IB, Montoya R, Moura MS, de Lacerda MV. [Malaria in Cruzeiro do Sul (Western Brazilian Amazon): analysis of the historical series from 1998 to 2008]. Rev Panam Salud Publica. 2010 Nov;28(5):353-60. PubMed PMID:21308180. 21308180 10.1590/s1020-49892010001100005

[6] Oliveira-Ferreira J, Lacerda MV, Brasil P, Ladislau JL, Tauil PL, Daniel-Ribeiro CT. Malaria in Brazil: an overview. Malar. J. 2010 Apr 30;9:115. doi:10.1186/1475-2875-9-115. 10.1186/1475-2875-9-115 PMC2891813 20433744

[7] WHO. World Malaria Report. 2016

[8] Vitor-Silva S, Siqueira AM, de Souza Sampaio V, Guinovart C, Reyes-Lecca RC, de Melo GC, et al. Declining malaria transmission in rural Amazon: changing epidemiology and challenges to achieve elimination. Malar. J. 2016;15:266 10.1186/s12936-016-1326-2 PMC4863332 27165432

[9] Ministério da Saúde. Programa Nacional de Prevenção e Controle da Malária - PNCM. 2003.

[10] Griffing SM, Tauil PL, Udhayakumar V, Silva-Flannery L. A historical perspective on malaria control in Brazil. Mem Inst Oswaldo Cruz. 2015 Sep;110(6):701-18. PubMed PMID:26517649. 26517649 10.1590/0074-02760150041 PMC4667572

[11] United Nations Brasil. OMS, Brasil e parceiro lançam plano de eliminação da malária no país. [cited: 2018 Fev 22]

[12] WHO. Global technical strategy for malaria 2016-2030. WHO Geneva 2015;1–35.

[13] UNDP. Sustainable Development Goals. 2018 [cited 2018 Feb 22]

[14] Castillo-Salgado C. Epidemiological risk sratification of malaria in the Américas. Mem. Inst. Oswaldo Cruz. 1992;87:115–20. 10.1590/s0074-02761992000700017 1343679

[15] Castillo-Salgado C, Bayona Celis M. Use of epidemiological research in the structuring epidemiological risk strata and the selection of control interventions. Washington DC: 2012.

[16] Alimi TO, Fuller DO, Herrera SV, Arevalo-Herrera M, Quinones ML, Stoler JB, Beier JC. A multi-criteria decision analysis approach to assessing malaria risk in northern South America. BMC Public Health. 2016 Mar 3;16:221. PubMed PMID:26940004. 26940004 10.1186/s12889-016-2902-7 PMC4778356

[17] Moreira Braz R, Luiz Tauil P, Carolina Faria e Silva Santelli A, Jesus Fernandes Fontes C. Avaliação da completude e da oportunidade das notificações de malária na Amazônia Brasileira, 2003-2012. Epidemiol. e Serviços Saúde 2016;25:10–1. 10.5123/S1679-49742016000100003 27861675

[18] WHO. World Malaria Report. 2015.

[19] Lima ID, Lapouble OM, Duarte EC. Time trends and changes in the distribution of malaria cases in the Brazilian Amazon Region, 2004-2013. Mem Inst Oswaldo Cruz. 2017 Jan 1;112(1):8-18. PubMed PMID:27925018. 27925018 10.1590/0074-02760160263 PMC5224350

[20] Recht J, Siqueira AM, Monteiro WM, Herrera SM, Herrera S, Lacerda MVG. Malaria in Brazil, Colombia, Peru and Venezuela: current challenges in malaria control and elimination. Malar J. 2017 Jul 4;16(1):273. PubMed PMID:28676055. 28676055 10.1186/s12936-017-1925-6 PMC5496604

[21] Rabinovich RN, Drakeley C, Djimde AA, Hall BF, Hay SI, Hemingway J, Kaslow DC, Noor A, Okumu F, Steketee R, Tanner M, Wells TNC, Whittaker MA, Winzeler EA, Wirth DF, Whitfield K, Alonso PL. malERA: An updated research agenda for malaria elimination and eradication. PLoS Med. 2017 Nov;14(11):e1002456. PubMed PMID:29190300. 29190300 10.1371/journal.pmed.1002456 PMC5708604

[22] malERA: An updated research agenda for characterising the reservoir and measuring transmission in malaria elimination and eradication. PLoS Med. 2017 Nov;14(11):e1002452. PubMed PMID:29190279. 29190279 10.1371/journal.pmed.1002452 PMC5708619

[23] Moonen B, Cohen JM, Snow RW, Slutsker L, Drakeley C, Smith DL, Abeyasinghe RR, Rodriguez MH, Maharaj R, Tanner M, Targett G. Operational strategies to achieve and maintain malaria elimination. Lancet. 2010 Nov 6;376(9752):1592-603. PubMed PMID:21035841. 21035841 10.1016/S0140-6736(10)61269-X PMC3037542

[24] Gomes EC de S, Albuquerque CMR de, Souza JRB de, Arruda ME, Confalonieri UEC. Structure of Anopheles (Diptera: Culicidae) population in areas with different degrees of human settlement: Cantá - Roraima - Brazil. Acta Amaz. 2008;38:321–9.

[25] Canelas T, Castillo-Salgado C, Ribeiro H. Systematized Literature Review on Spatial Analysis of Environmental Risk Factors of Malaria Transmission. Adv. Infect. Dis. 2016;6:52–62

[26] Stresman GH. Beyond temperature and precipitation: ecological risk factors that modify malaria transmission. Acta Trop. 2010 Dec;116(3):167-72. PubMed PMID:20727338. 20727338 10.1016/j.actatropica.2010.08.005

[27] Weiss DJ, Mappin B, Dalrymple U, Bhatt S, Cameron E, Hay SI, Gething PW. Re-examining environmental correlates of Plasmodium falciparum malaria endemicity: a data-intensive variable selection approach. Malar J. 2015 Feb 7;14:68. PubMed PMID:25890035. 25890035 10.1186/s12936-015-0574-x PMC4333887

[28] Reis IC, Honório NA, Barros FS, Barcellos C, Kitron U, Camara DC, Pereira GR, Keppeler EC, da Silva-Nunes M, Codeço CT. Epidemic and Endemic Malaria Transmission Related to Fish Farming Ponds in the Amazon Frontier. PLoS One. 2015;10(9):e0137521. PubMed PMID:26361330. 26361330 10.1371/journal.pone.0137521 PMC4567347

[29] Ferreira MU, Da Silva-Nunes M. Evidence-based public health and prospects for malaria control in Brazil. J Infect Dev Ctries. 2010 Oct 4;4(9):533-45. PubMed PMID:21045365. 21045365 10.3855/jidc.760

[30] Vieira Gde D, Basano Sde A, Katsuragawa TH, Camargo LM. Insecticide-treated bed nets in Rondônia, Brazil: evaluation of their impact on malaria control. Rev Inst Med Trop Sao Paulo. 2014 Nov-Dec;56(6):493-7. PubMed PMID:25351543. 25351543 10.1590/S0036-46652014000600007 PMC4296869

[31] Martins-Campos KM, Pinheiro WD, Vítor-Silva S, Siqueira AM, Melo GC, Rodrigues IC, Fé NF, Barbosa Md, Tadei WP, Guinovart C, Bassat Q, Alonso PL, Lacerda MV, Monteiro WM. Integrated vector management targeting Anopheles darlingi populations decreases malaria incidence in an unstable transmission area, in the rural Brazilian Amazon. Malar J. 2012 Oct 23;11:351. PubMed PMID:23088224. 23088224 10.1186/1475-2875-11-351 PMC3502175

[32] Sinka ME, Bangs MJ, Manguin S, Coetzee M, Mbogo CM, Hemingway J, Patil AP, Temperley WH, Gething PW, Kabaria CW, Okara RM, Van Boeckel T, Godfray HC, Harbach RE, Hay SI. The dominant Anopheles vectors of human malaria in Africa, Europe and the Middle East: occurrence data, distribution maps and bionomic précis. Parasit Vectors. 2010 Dec 3;3:117. PubMed PMID:21129198. 21129198 10.1186/1756-3305-3-117 PMC3016360

[33] Campos M, Conn JE, Alonso DP, Vinetz JM, Emerson KJ, Ribolla PE. Microgeographical structure in the major Neotropical malaria vector Anopheles darlingi using microsatellites and SNP markers. Parasit Vectors. 2017 Feb 13;10(1):76. PubMed PMID:28193289. 28193289 10.1186/s13071-017-2014-y PMC5307779

[34] Lourenço-de-Oliveira R, Guimarães AE, Arlé M, da Silva TF, Castro MG, Motta MA, Deane LM. Anopheline species, some of their habits and relation to malaria in endemic areas of Rondônia State, Amazon region of Brazil. Mem Inst Oswaldo Cruz. 1989 Oct-Dec;84(4):501-14. PubMed PMID:2487447. 2487447 10.1590/s0074-02761989000400008

[35] Braz RM, Guimarães RF, Carvalho Júnior OA, Tauil PL. Spatial dependence of malaria epidemics in municipalities of the Amazonian Ecosystem. Rev Bras Epidemiol. 2014 Jul-Sep;17(3):615-28. PubMed PMID:25272256. 25272256 10.1590/1809-4503201400030004

[36] de Castro MC, Monte-Mór RL, Sawyer DO, Singer BH. Malaria risk on the Amazon frontier. Proc Natl Acad Sci U S A. 2006 Feb 14;103(7):2452-7. PubMed PMID:16461902. 16461902 10.1073/pnas.0510576103 PMC1413719

[37] Observatorio Venezolano de la Salud. OVS | Epidemia de malaria, sin control ni medicamentos, puede llegar a 350 mil casos al finalizar 2016. 2016

[38] Pan American Health Organization. Epidemiological Alert Increase in cases of malaria. Washington DC: 2017

[39] PAHO. Transcending politics and using evidence-based treatment policies and public health approaches in combating malaria : The Amazon Malaria Initiative ( AMI ) and the Amazon Network for the Surveillance of Anti- malarial Drug Resistance ( RAVREDA ) Partnersh. 2007

[40] Howes RE, Battle KE, Mendis KN, Smith DL, Cibulskis RE, Baird JK, Hay SI. Global Epidemiology of Plasmodium vivax. Am J Trop Med Hyg. 2016 Dec 28;95(6 Suppl):15-34. PubMed PMID:27402513. 27402513 10.4269/ajtmh.16-0141 PMC5198891

[41] Barbosa S, Gozze AB, Lima NF, Batista CL, Bastos Mda S, Nicolete VC, Fontoura PS, Gonçalves RM, Viana SA, Menezes MJ, Scopel KK, Cavasini CE, Malafronte Rdos S, da Silva-Nunes M, Vinetz JM, Castro MC, Ferreira MU. Epidemiology of disappearing Plasmodium vivax malaria: a case study in rural Amazonia. PLoS Negl Trop Dis. 2014 Aug;8(8):e3109. PubMed PMID:25166263. 25166263 10.1371/journal.pntd.0003109 PMC4148206

[42] Cheng Q, Cunningham J, Gatton ML. Systematic review of sub-microscopic P. vivax infections: prevalence and determining factors. PLoS Negl Trop Dis. 2015 Jan;9(1):e3413. PubMed PMID:25569135. 25569135 10.1371/journal.pntd.0003413 PMC4288718

[43] Alexandre MA, Ferreira CO, Siqueira AM, Magalhães BL, Mourão MP, Lacerda MV, Alecrim Md. Severe Plasmodium vivax malaria, Brazilian Amazon. Emerg Infect Dis. 2010 Oct;16(10):1611-4. PubMed PMID:20875292. 20875292 10.3201/eid1610.100685 PMC3294402

[44] Marques MM, Costa MR, Santana Filho FS, Vieira JL, Nascimento MT, Brasil LW, Nogueira F, Silveira H, Reyes-Lecca RC, Monteiro WM, Lacerda MV, Alecrim MG. Plasmodium vivax chloroquine resistance and anemia in the western Brazilian Amazon. Antimicrob Agents Chemother. 2014;58(1):342-7. PubMed PMID:24165179. 24165179 10.1128/AAC.02279-12 PMC3910749

[45] Gonçalves LA, Cravo P, Ferreira MU. Emerging Plasmodium vivax resistance to chloroquine in South America: an overview. Mem Inst Oswaldo Cruz. 2014 Aug;109(5):534-9. PubMed PMID:25184999. 25184999 10.1590/0074-0276130579 PMC4156446

[46] Valle D, Lima JM. Large-scale drivers of malaria and priority areas for prevention and control in the Brazilian Amazon region using a novel multi-pathogen geospatial model. Malar J. 2014 Nov 20;13:443. PubMed PMID:25412882. 25412882 10.1186/1475-2875-13-443 PMC4247612

[47] Kohara Melchior LA, Chiaravalloti Neto F. Spatial and spatio-temporal analysis of malaria in the state of Acre, western Amazon, Brazil. Geospat Health. 2016 Nov 16;11(3):443. PubMed PMID:27903051. 27903051 10.4081/gh.2016.443

[48] Lana RM, Riback TIS, Lima TFM, da Silva-Nunes M, Cruz OG, Oliveira FGS, Moresco GG, Honório NA, Codeço CT. Socioeconomic and demographic characterization of an endemic malaria region in Brazil by multiple correspondence analysis. Malar J. 2017 Oct 2;16(1):397. PubMed PMID:28969634. 28969634 10.1186/s12936-017-2045-z PMC5625626

[49] Wilson ML, Krogstad DJ, Arinaitwe E, Arevalo-Herrera M, Chery L, Ferreira MU, Ndiaye D, Mathanga DP, Eapen A. Urban Malaria: Understanding its Epidemiology, Ecology, and Transmission Across Seven Diverse ICEMR Network Sites. Am J Trop Med Hyg. 2015 Sep;93(3 Suppl):110-23. PubMed PMID:26259941. 26259941 10.4269/ajtmh.14-0834 PMC4574269

